# Biallelic variants in *COX18* cause a mitochondrial disorder primarily manifesting as peripheral neuropathy

**DOI:** 10.1093/brain/awaf300

**Published:** 2025-08-20

**Authors:** Camila Armirola-Ricaurte, Laura Morant, Isabelle Adant, Sherifa A Hamed, Menelaos Pipis, Stephanie Efthymiou, Silvia Amor-Barris, Derek Atkinson, Liedewei Van de Vondel, Aleksandra Tomic, Sara Seneca, Els de Vriendt, Stephan Zuchner, Bart Ghesquiere, Michael G Hanna, Henry Houlden, Michael P Lunn, Mary M Reilly, Vedrana Milic Rasic, Albena Jordanova

**Affiliations:** Molecular Neurogenomics Group, VIB Center for Molecular Neurology, VIB, Antwerp 2610, Belgium; Molecular Neurogenomics Group, Department of Biomedical Sciences, University of Antwerp, Antwerp 2610, Belgium; Molecular Neurogenomics Group, VIB Center for Molecular Neurology, VIB, Antwerp 2610, Belgium; Molecular Neurogenomics Group, Department of Biomedical Sciences, University of Antwerp, Antwerp 2610, Belgium; Laboratory of Hepatology, Department of Chronic Diseases, Metabolism and Ageing, Katholieke Universiteit Leuven, Leuven 3000, Belgium; Department of Pediatrics, University Hospitals Leuven, Leuven 3000, Belgium; Department of Neurology and Psychiatry, Assiut University Hospitals, Assiut 2074020, Egypt; Centre for Neuromuscular Diseases, Department of Neuromuscular Diseases, UCL Queen Square Institute of Neurology, London WC1N 3BG, UK; Neuropathology Department and Center for Neuromuscular Disorders, The Cyprus Institute of Neurology and Genetics, Nicosia 2371, Cyprus; Centre for Neuromuscular Diseases, Department of Neuromuscular Diseases, UCL Queen Square Institute of Neurology, London WC1N 3BG, UK; Molecular Neurogenomics Group, VIB Center for Molecular Neurology, VIB, Antwerp 2610, Belgium; Molecular Neurogenomics Group, Department of Biomedical Sciences, University of Antwerp, Antwerp 2610, Belgium; Molecular Neurogenomics Group, VIB Center for Molecular Neurology, VIB, Antwerp 2610, Belgium; Molecular Neurogenomics Group, Department of Biomedical Sciences, University of Antwerp, Antwerp 2610, Belgium; Translational Neurosciences, Faculty of Medicine and Health Sciences, University of Antwerp, Antwerp 2610, Belgium; Laboratory of Neuromuscular Pathology, Institute Born-Bunge, University of Antwerp, Antwerp 2610, Belgium; Faculty of Medicine, University of Belgrade, Belgrade 11000, Serbia; Neurology Clinic, University Clinical Center of Serbia, Belgrade 11000, Serbia; Clinical Sciences, Research Group Reproduction and Genetics, Centre for Medical Genetics, Universitair Ziekenhuis Brussel (UZ Brussel), Vrije Universiteit Brussel (VUB), Brussels 1090, Belgium; Molecular Neurogenomics Group, VIB Center for Molecular Neurology, VIB, Antwerp 2610, Belgium; Molecular Neurogenomics Group, Department of Biomedical Sciences, University of Antwerp, Antwerp 2610, Belgium; Dr. John T. Macdonald Foundation Department of Human Genetics, University of Miami Miller School of Medicine, Miami, FL 33136, USA; John P. Hussman Institute for Human Genomics, University of Miami Miller School of Medicine, Miami, FL 33136, USA; Metabolomics Expertise Center, Center for Cancer Biology, CCB-VIB, Leuven 3000, Belgium; Metabolomics Expertise Center, Department of Oncology, Katholieke Universiteit Leuven, Leuven 3000, Belgium; Centre for Neuromuscular Diseases, Department of Neuromuscular Diseases, UCL Queen Square Institute of Neurology, London WC1N 3BG, UK; Centre for Neuromuscular Diseases, Department of Neuromuscular Diseases, UCL Queen Square Institute of Neurology, London WC1N 3BG, UK; Centre for Neuromuscular Diseases, Department of Neuromuscular Diseases, UCL Queen Square Institute of Neurology, London WC1N 3BG, UK; Centre for Neuromuscular Diseases, Department of Neuromuscular Diseases, UCL Queen Square Institute of Neurology, London WC1N 3BG, UK; Faculty of Medicine, University of Belgrade, Belgrade 11000, Serbia; Clinic for Neurology and Psychiatry for Children and Youth, Belgrade 11000, Serbia; Molecular Neurogenomics Group, VIB Center for Molecular Neurology, VIB, Antwerp 2610, Belgium; Molecular Neurogenomics Group, Department of Biomedical Sciences, University of Antwerp, Antwerp 2610, Belgium; Department of Medical Chemistry and Biochemistry, Medical University-Sofia, Sofia 1431, Bulgaria

**Keywords:** cytochrome *c* oxidase assembly factor 18, complex IV deficiency, Charcot–Marie–Tooth disease

## Abstract

Defects in mitochondrial dynamics are a common cause of Charcot–Marie–Tooth disease (CMT), whereas primary deficiencies in the mitochondrial respiratory chain (MRC) are rare and atypical for this aetiology. This study aims to report *COX18* as a novel CMT-causing gene. This gene encodes an assembly factor of mitochondrial Complex IV that translocates the C-terminal tail of MTCO2 across the mitochondrial inner membrane.

Exome sequencing was performed in four affected individuals from three families. The patients and available family members underwent thorough neurological and electrophysiological assessment. The impact of one of the identified variants on splicing, protein levels and mitochondrial bioenergetics was investigated in patient-derived lymphoblasts. The functionality of the mutant protein was assessed using a proteinase K protection assay and immunoblotting. Neuronal relevance of COX18 was assessed in a *Drosophila melanogaster* knockdown model.

Exome sequencing coupled with homozygosity mapping revealed a homozygous splice variant c.435–6A>G in *COX18* in two siblings with early-onset progressive axonal sensorimotor peripheral neuropathy. By querying external databases, we identified two additional families with rare deleterious biallelic variants in *COX18*. All eight affected individuals presented with axonal CMT, and some patients also exhibited CNS symptoms, such as dystonia and spasticity. Functional characterization of the c.435-6A>G variant demonstrated that it leads to the expression of an alternative transcript that lacks exon 2, resulting in a stable but defective COX18 isoform. The mutant protein impairs Complex IV assembly and activity, leading to a reduction in mitochondrial membrane potential. Downregulation of the *COX18* homologue in *D. melanogaster* resulted in signs of neurodegeneration, including locomotor deficit and progressive axonal degeneration of sensory neurons.

Our study presents genetic and functional evidence that supports *COX18* as a newly identified gene candidate for autosomal recessive axonal CMT with or without CNS involvement. These findings emphasize the significance of peripheral neuropathy within the spectrum of primary mitochondrial disorders, in addition to the role of mitochondrial Complex IV in the development of CMT. Our research has important implications for the diagnostic work-up of CMT patients.

## Teaser

Armirola-Ricaurte *et al.* report that variants in the *COX18* gene can cause autosomal recessive axonal Charcot-Marie-Tooth disease type 2 with or without CNS involvement. Functional studies suggest that partial loss of function of COX18 impairs assembly and activity of mitochondrial complex IV.

## Introduction

Charcot–Marie–Tooth (CMT) disease refers to a group of clinically and genetically heterogeneous sensorimotor peripheral neuropathies. Together, they are the most common inherited neuromuscular disorder, with a prevalence of 9.7–82.3 patients per 100 000 individuals.^1^ More than 100 genes across all patterns of inheritance have been linked to CMT.^2^ Some of these genes encode proteins that participate in essential nerve-specific processes, such as axonal transport, myelination and synaptic transmission. Others are involved in general housekeeping pathways (e.g. endosomal trafficking, mRNA processing). It remains unclear why defects in such ubiquitous proteins predominantly affect peripheral nerves.

Mitochondrial dynamics plays a significant role in CMT.^3^ Variants in *MFN2*, involved in mitochondrial fusion, are among the most prevalent causes of axonal CMT (CMT2), accounting for 21%–30% of the genetically diagnosed individuals.^4,5^ Likewise, pathogenic variants in *SLC25A46*, another mitochondrial fusion factor, have been linked to CMT.^6^ Variants in *GDAP1*, encoding a protein implicated in mitochondrial fission, are another common cause of CMT2, specifically prevalent in certain geographical regions of the world.^7-10^ Defects in these proteins impair mitochondrial trafficking, distribution and turnover along the axons, ultimately leading to bioenergetic failure.^11,12^

Although mitochondrial dynamics defects are a well-established pathway in CMT, primary defects in the respiratory chain are a rare and relatively unexplored aetiology. In the past decade, next-generation sequencing studies have demonstrated that defects in the components or assembly of almost every mitochondrial complex can lead to peripheral neuropathy as a predominant manifestation. These include biallelic pathogenic variants in genes encoding structural subunits of Complex I (*NDUFS6*, *NDUFA9* and *MT-ND3*) or genes encoding subunits and assembly factors of cytochrome *c* oxidase (Complex IV) (*SURF1*, *COA7*, *COX6A1* and *COX20*).^13-22^ Finally, a variant in the mitochondrially encoded MT-ATP6, a subunit of ATP synthase (Complex V), has been estimated to account for ∼1% of unsolved CMT2 patients.^23^

Here, we report biallelic variants in *COX18* (cytochrome *c* oxidase assembly factor 18), as a novel cause of CMT2. Defects in this gene have very recently been associated with severe and rapidly progressive encephalopathy with neonatal or infantile onset and associated with cardiomyopathy, oculomotor apraxia and peripheral neuropathy.^24,25^ In the present study, patients carrying deleterious variants in *COX18* exhibit as a cardinal feature progressive sensorimotor polyneuropathy of variable onset, and some of them also display signs of CNS involvement. Functional studies in patient-derived lymphoblasts suggest that the underlying mechanism is a partial loss of function of COX18 that leads to reduced assembly and activity of Complex IV (CIV). Downregulation of the orthologue of COX18 in *Drosophila* induces behavioural and neuropathological phenotypes common to other fly models of CMT disease.

## Materials and methods

### Participants

Patients underwent routine neurological and electrophysiological examinations. This study was approved by the local institutional review boards. All patients signed an informed consent form before enrolment.

### Exome sequencing and homozygosity mapping

Genomic DNA was isolated from peripheral blood mononuclear cells according to standard protocols. Exome sequencing (ES) was performed in the two affected members from Family 1 using SeqCap EZ Exome Probes v.3.0 kit (Roche) for capture. Then, 150 bp exome paired-end sequencing was run on a NextSeq 150 platform (Illumina). Sequencing read mapping, variant calling and annotation were done using GenomeComb (v.0.98.3).^26^ Homozygosity mapping based on ES data was performed using the HOMWES tool as described.^27^ Variants were filtered and prioritized within the resulting regions of homozygosity based on a recessive model of inheritance with the following criteria: non-synonymous or splice variants with minor allele frequency (MAF) <5% and no homozygotes in the control population database gnomAD v.2.1.1.^28^ Prioritized variants were evaluated further using Alamut Visual Plus (Sophia Genetics).

### Mitochondrial DNA sequencing and analysis

DNA of the proband from Family 1 (1.II.1) was isolated from peripheral blood mononuclear cells and the mitochondrial DNA (mtDNA) was enriched using long-range PCR. A DNA library was prepared using the NebNext kit (Bioké) and, 2 × 100 bp paired-end sequencing was performed on a NovaSeq 6000 machine (Illumina). Additionally, the mtDNA sequencing reads from the probands of Families 2 (2.II.1) and 3 (3.II.1) were derived from the ES data available. The reads were aligned to the human mitochondrial reference genome, NC_012920.1. After standard quality control, variant calling and annotation, variants were annotated with MToolBox^29^ and MITOMAP.^30^ Haplogroup was determined with Haplogrep v.2.4.0.^31^ The sequencing data were analysed and interpreted based on available information in the literature and published databases, including gnomAD^28^ and MITOMAP.^30^

### Variant segregation and cohort screening

The resulting variants were confirmed and segregated in the available family members by Sanger sequencing, as previously described.^27^ A cohort of 362 CMT patients with unknown genetic diagnosis was screened for deleterious variants in *COX18* using an amplicon target amplification assay (Agilent; https://www.agilent.com). Resequencing was performed on a MiSeq (Illumina) platform using 250 bp paired-end reads targeting all exons and exon–intron boundaries of the canonical *COX18* transcripts. The primers used are listed in Supplementary Table 1. All additional variants identified in the cohort screening were validated by Sanger sequencing. Furthermore, GENESIS^32^ and RD-Connect Genome-Phenome Analysis Platform (GPAP) (https://platform.rd-connect.eu/)^33^ were queried online to find additional unrelated CMT patients with *COX18* candidate variants. In this way, two additional COX18 families were identified (Family 2 via GPAP and Family 3 via GENESIS).

### Cell culture

Peripheral blood mononuclear cells from the patients and parents of Family 1 were isolated and transformed with Epstein–Barr virus (EBV) as described.^34^

### RNA extraction, cDNA synthesis and RT-qPCR

Total RNA was extracted from the lymphoblasts using the Universal RNA Purification kit (Roboklon), followed by DNAse treatment with the TURBO DNA-free kit (Invitrogen, Thermo Fisher Scientific). Complementary DNA was synthesized using iScript Advanced cDNA synthesis kit for RT-qPCR (Bio-Rad). *COX18* cDNA was amplified using the primers listed in Supplementary Table 1. *COX18* transcripts of interest were amplified using the Power SYBR green PCR master mix (Applied Biosystems), and the fluorescence was measured in the the QuantStudio™ 6 Flex Real-Time PCR System (Thermo Fisher Scientific). Gene expression levels were measured using Qbase+ software (Biogazelle). Five different housekeeping genes (*GAPDH*, *SDHA*, *TBP*, *HPRT1* and *HMBS*) were included in the analysis to normalize the data across the samples. The primers used for RT-qPCR are listed in Supplementary Table 1.

### Targeted cDNA long-read sequencing

Targeted long-read sequencing (T-LRS) of *COX18* cDNA was performed on a MinION platform using a Flongle adapter (Oxford Nanopore Technologies) as described.^35^ For this purpose, a primer pair was designed to anneal on exon 1 and 4, flanking all previously known splicing events of *COX18*. Given that *COX18* is expressed at low levels in most tissues, PCR amplification with 35 cycles was performed. Sequencing reads were aligned to human genome assembly 38 with minimap2 v.5.0.11 in spliced alignment mode. Read splice junction correction, high-confidence isoform definition and quantification were performed using FLAIR v.1.5.^36^

### Mitochondrial isolation

Lymphoblasts were collected from one T175 flask per individual, washed with PBS, and resuspended in mitochondrial isolation buffer (250 mM mannitol, 0.5 mM EGTA and 5 mM HEPES/KOH, pH 7.4). The cells were lysed using 10 strokes of a 1 ml syringe attached to a 26.5 gauge needle. The mitochondrial fractions were isolated by differential centrifugation as described.^37^

### Proteinase K protection assay

Proteinase K protection assay was conducted as described.^37^ Mitochondria were divided equally into four tubes, two of which were resuspended in mitochondrial isolation buffer and two in osmotic swelling buffer without mannitol (0.5 mM EGTA and 5 mM HEPES/KOH, pH 7.4) to generate mitoplasts. One tube from each condition was treated with 5 μg/ml of proteinase K (Thermo Fisher Scientific) for 20 min at 4°C. Digestion was inhibited by incubation with 1 mM phenylmethylsulphonyl fluoride solution (sc-482875, Santa Cruz Biotechnology) for 10 min on ice. Mitochondria and mitoplasts were isolated by centrifugation (10 000*g*, 10 min, 4°C), resuspended in Laemmli buffer, boiled for 5 min, and immunoblotted as explained below.

### Complex IV enzymatic assay

Complex IV activity was measured in mitochondrial fractions as previously described.^38^ Lymphoblast pellets were obtained from one T175 flask and resuspended in 1 ml ice-cold Mega Fb buffer (250 mM sucrose, 2 mM HEPES and 0.1 mM EGTA, pH 7.4) supplemented with 0.08 mM digitonin. The cells were disrupted on an ice slurry with 20 strokes of a Teflon-glass Wheaton homogenizer driven by a Glas-Col High Speed Homogenizer variable speed bench top drill at 1800 rpm. Mitochondrial fractions were isolated by differential centrifugation as described.^37^ The resulting pellet was resuspended in ice-cold hypotonic buffer (25 mM potassium phosphate, pH 7.2, and 5 mM MgCl_2_) and subjected to three freeze–thaw cycles in dry ice and ethanol slurry. CIV and citrate synthase (CS) activity were measured in a Cary 300 UV-Vis spectrophotometer (model number G9823A) as previously described.^39^ Before measurements, reaction cuvettes with 50 mM KPi (pH 7.4) were equilibrated to 30°C. Each enriched mitochondria sample was added, followed by the addition of reduced cytochrome *c* to a concentration of 15 mM. After briefly mixing, the absorbance was measured immediately. Then, K_3_Fe(CN)_6_ was added to 1 mM to achieve complete oxidation of cytochrome *c*, and a final reading was taken. The results were calculated as described.^39^

### Measurement of mitochondrial membrane potential

Lymphoblasts were washed twice with prewarmed PBS and incubated with 20 μM TMRE (ENZ-52309, tetramethylrhodamine ethyl ester perchrolate, Enzo Life Sciences) for 30 min at 37°C. Positive control cells were incubated with 20 nM FCCP (ab120081, Abcam) for 5 min at 37°C before staining. After staining, cells were rinsed with prewarmed PBS and analysed with flow cytometry on a MACSQuant Analyzer 10 (Miltenyi Biotec). Median fluorescence intensity (MFI) was measured using FlowLogic v.8.6 software (Inivai Technologies, Mentone, Victoria, Australia).

### Western blotting

Total protein or mitochondrial fractions from lymphoblasts were lysed, separated by SDS-PAGE and transferred to blotting membranes as described.^34^ Membranes were incubated with the following primary antibodies: anti-COX18 (Protein atlas, HPA049489, 1:1000), anti-MTCO2 (Abcam, ab913117, 1:1000), anti-α-tubulin (Abcam, ab7291, 1:5000), anti-VDAC1 (Abcam, ab14734,1:1000), anti-SDHA (Genetex, GTX632636, 1:3000) anti-HCCS (Proteintech, 15118-1-AP, 1:2000), OXPHOS Human WB Antibody cocktail (Abcam, ab110411, 1:200) and anti-ATP5C1 (ThermoFisher Scientific, 60284-1-IG, 1:1000). Anti-rabbit IgG (Promega, W401B, 1:10 000) and anti-mouse IgG1 and IgG2b (Southern Biotech, 1070-05 and 1090-05, 1:10 000) were used as secondary antibodies. The blots were developed with Pierce ECL Plus substrate (Thermo Fisher Scientific) and imaged using the Amersham Imager 600 (GE Healthcare). Images were analysed using ImageJ^40^ to calculate the mean pixel grey values of each band.

### *Drosophila* stocks and maintenance

The following fly stocks were obtained from the Bloomington Drosophila Stock Center (Bloomington, IN, USA): UAS-mCD8::ChRFP (BL27391), dpr-Gal4 (BL25083) and *Mi{MIC}CG4942^MI03165^* (d*COX18^MI03165^*, BL36211). The RNA interference (RNAi) lines for d*COX18* (RNAi-d*COX18*, *CG4942^GD2380^*, v42888) and Sply (RNAi-*Sply*, *Sply^HMS02526^*, v42834) were obtained from Vienna Drosophila Resource Center (Vienna, Austria). The UAS-*YARS1* fly line expressing human YARS1 with a CMT-causing variant (*YARS1-E196K*) was generated and described previously.^41^ The nSyb-Gal4 driver line was kindly provided by M. Leyssen and B. Dickson.^42^ All crosses were performed at 25°C, 12 h–12 h light–dark cycle, on Nutri-Fly™ flood (Flystuff, San Diego, CA, USA).

### Fly RNA extraction, cDNA synthesis and RT-qPCR

Total RNA was isolated from the heads of adult flies following the standard Trizol (Qiagen) and chloroform extraction protocol. RNA was treated with TURBO DNA-free kit (Invitrogen, Thermo Fisher Scientific) followed by cDNA synthesis using iScript Advanced cDNA synthesis kit for RT-qPCR (Bio-Rad). To confirm the downregulation of the orthologue of *COX1*8, d*COX18* (*CG4942*), in the heterozygous d*COX18^MI03165^* fly line, gene expression levels were determined by quantitative RT-PCR using SYBR green PCR master mix (Applied Biosystems) and compared with nSyb-GAL4-driven d*COX18* pan-neuronal knockdown using RNAi (nSyb-GAL4>RNAi-d*COX18*) and a control *yw* fly line. The following targets were used as housekeeping genes: *Gapdh1*, *RpL32*, *RpS13*, *Act42A*, *Act79B*. The housekeeping gene with the most stable expression across samples was used to normalize the data. The results were analysed using the qBase+ software. The primers used for RT-qPCR are described in Supplementary Table 1.

### Wing degeneration assay

A *Drosophila* wing degeneration assay was performed as described previously.^43^ Flies carrying the UAS-mCD8::ChRFP construct in their genome were crossed with flies with the dpr-GAL4 driver to express the mChrerry protein in the chemosensory neurons of the wing margin bristles. The construct encodes a membrane-bound RFP-labelled mCherry protein that is incorporated into the neuronal membrane. When the flies reached the desired age (1 or 30 days post-eclosion), one wing per fly was clipped as close as possible to the thorax. Each wing was washed with PBS supplemented with 0.2% Triton X-100, then mounted in Dako mounting medium (Agilent Technologies). Axonal degeneration was determined by visual inspection of the fragmentation of the neuronal membrane, and the proportion of wings depicting the fragmented phenotype per fly line was calculated.

### Negative geotaxis assay

The effect of d*COX18* downregulation on fly locomotion was studied using a negative geotaxis assay as described.^44^ From each fly line, 10 days post-eclosion female flies with clipped wings were placed in a closed vial 49 mm in diameter. Fly movements were recorded using an infrared camera. After acclimation for 1 h, the flies were tapped onto the bottom of the vial using a semi-automated FlyCrawler device (Peira Scientific Instruments). For each genotype, 10 groups of 10 flies were tested ≥15 times, and the average time taken by the 10 fastest flies to reach a mark at a height of 82 mm was calculated.

### Statistical analyses

GraphPad Prism v.9.2.0 was used for statistical analyses. The test for each experimental measurement is reported within the figure legends. Briefly, continuous variables were compared with Student's two-tailed *t*-test or a one-way ANOVA followed by Tukey's multiple-comparison test. Categorical variables were analysed by pairwise comparison with a one-sided χ^2^ test. In all figures, *P*-values are reported as follows: *P < 0.05, **P < 0.01, ***P < 0.001, ****P < 0.0001, and ns = non-significant.

## Results

### Exome sequencing identifies three independent families with biallelic deleterious variants in *COX18*

**Figure 1:**
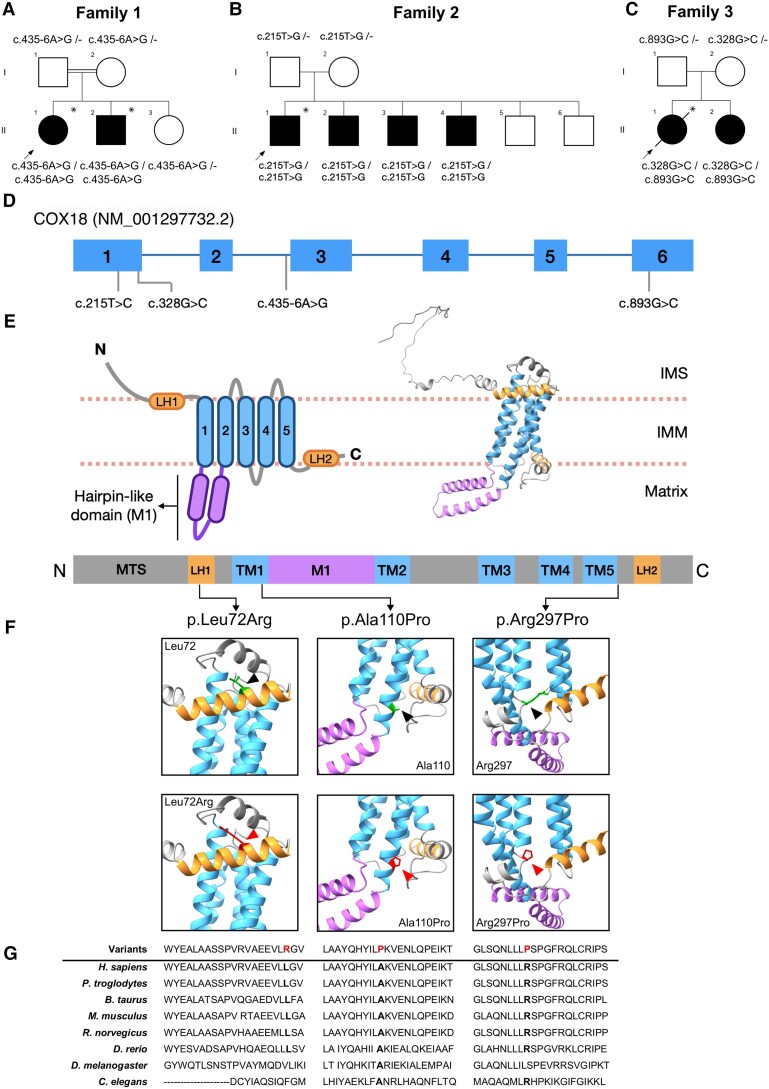
**Biallelic variants in *COX18* in three families with autosomal recessive axonal CMT.** (**A**–**C**) Pedigree and variant segregation of the Families 1–3. Squares indicate males and circles represent females. Solid symbols indicate affected individuals. Arrows point to the proband of each family. Asterisks specify the patients whose exome was sequenced. The diagonal line across a symbol denotes a deceased individual. (**D**) Distribution of the variants on the *COX18* gene. (**E**) Diagram of COX18 domains (*top left*), protein structure model of COX18 from AlphaFold ^49,50^ (*top right*) and distribution of the variants on the COX18 protein (*bottom*). (**F**) The position of the residues affected by the missense variants is shown within 3D protein structure of COX18 predicted by AlphaFold.^49,50^ Original amino acids (green) are indicated by black arrowheads (*top row*), whereas mutated residues (red) are indicated by red arrowheads (*bottom row*). (**G**) Amino acid conservation of the Leu72, Ala110 and Arg297 residues across multiple species. IMM = inner mitochondrial membrane; IMS = intermembrane space; LH = amphipathic helix; MTS = mitochondrial targeting sequence; M1 = hairpin-like domain; TM = transmembrane helix.

**Figure 2:**
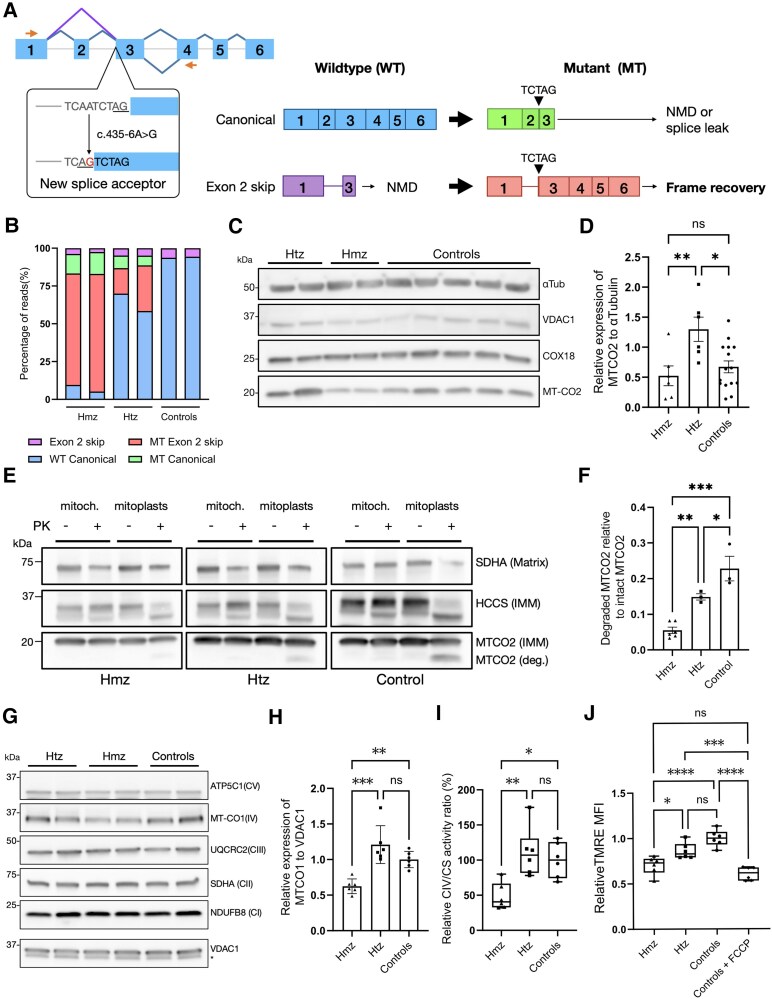
**Functional characterization of COX18:c.435–6A>G variant in patients’ EBV-transformed lymphoblasts.** (**A**) Exon–intron architecture of wild-type *COX18* transcripts. Splicing junctions are depicted as carets connecting the exons, junctions of the canonical transcripts (ENST00000507544.3 and ENST00000295890.8) are coloured in blue, and the exon 1-3 junction of the exon 2 skip transcript (ENST00000449739.6) is shown in purple. *Left*: The small orange-filled arrows represent the forward and reverse primers used for *COX18* cDNA targeted long-read sequencing (T-LRS). The effect of the variant on splicing is shown in the dialogue box below exon 3. *Right*: Diagrams of the wild-type transcripts and the abnormally spliced transcripts that result from the splice variant. (**B**) Relative quantification of *COX18* transcripts sequenced by cDNA T-LRS in homozygous (hmz), heterozygous (htz) and control individuals shows that the mutant transcript skipping exon 2 is the predominant transcript in the homozygous patients (*n* = 1 for each genotype, with two biological replicates for each genotype). (**C** and **D**) Immunoblotting of full lysate protein from EBV-transformed lymphoblasts derived from the individuals with different genotypes shows no reduced levels of COX18. Please note that mutant COX18 protein, resulting from the transcript with frame recovery, is predicted to be only 4 kDa smaller than wild-type COX18, making the two isoforms indistinguishable from each other on the blot. Western blot analysis revealed reduced levels of MTCO2 in the homozygotes compared with the carrier but not to five unrelated controls. Data are shown as the mean ± standard deviation (SD) (*n* = 3). (**E** and **F**) Proteinase K (PK) protection assay in mitoplasts from lymphoblasts of the homozygous proband indicates that MTCO2 is protected from degradation, whereas heterozygotes and unrelated controls show increased levels of degraded MTCO2. The levels of degraded MTCO2 (deg. MTCO2) relative to the levels of intact MTCO2 in the untreated mitochondrial fraction are shown as the mean ± SD (*n* = 3 for each genotype; two biological replicates were tested for the homozygous genotype). SDHA is a mitochondrial matrix protein and HCCS is an inner mitochondrial (IMM) protein. (**G** and **H**) Western blot of mitochondrial fractions from lymphoblasts revealed decreased protein levels of Complex IV (CIV) subunit MTCO1 in the homozygotes relative to unrelated controls and heterozygotes. The asterisk indicates an unspecific band. Immunoblotting quantification of MTCO1 is shown as mean ± SD (*n* = 3 for each genotype, with two biological replicates for each genotype). (**I**) Normalized CIV to citrate synthetase (CS) activity is decreased in homozygotes compared with the other genotypes. Values are shown as the mean ± SD (*n* = 3). (**J**) Flow cytometry analysis of mitochondrial membrane potential with TMRE staining shows decreased median fluorescence intensity (MFI) in lymphoblasts from homozygous patients relative to unrelated controls. ****P < 0.0001, ***P < 0.001, **P < 0.01, *P < 0.05, ns = not significant.

ES was performed on the affected siblings from Family 1. Initially, we screened for potential pathogenic variants in known CMT genes, but no causal variants were found. Given the reported consanguinity in the family, we then conducted a HOMWES analysis, which revealed 17 regions of homozygosity shared between both affected individuals, totalling 33.4 Mb in size (the biggest of 8 Mb). Variant filtering within those regions and prioritization based on impact and population frequency led to the identification of a homozygous splice variant, NM_001297732.2:c.435-6A>G, in the second intron of *COX18*. The splice variant was confirmed by Sanger sequencing, and analysis of available relatives showed that it co-segregated with the disease (Fig. 1A). The variant was extremely rare in the control population database gnomAD v.4.1.0,^45^ with an allele frequency of 0.00001 and no homozygotes. The variant was predicted by multiple *in silico* tools (SpliceSiteFinder-like,^46^ MaxEntScan^47^ and NNSPLICE^48^) to abolish the canonical acceptor site in exon 3 and to generate a new acceptor site 6 bp upstream in intron 2 (Fig. 2A). The resulting frameshift was therefore expected to create a premature stop codon in exon 3 and to cause nonsense-mediated decay of *COX18* canonical transcripts (Fig. 2A). Additionally, mtDNA sequencing showed that the proband from Family 1 carried the H1 haplogroup and no pathogenic mtDNA variants.

Screening for additional individuals with *COX18* biallelic deleterious variants in our in-house cohort of 362 unsolved CMT patients (272 with suggestive recessive inheritance and 90 sporadic) did not return any additional affected individuals. However, two additional families were found through the GENESIS^32^ and RD-Connect^33^ online platforms. Family 2 consisted of four siblings who harboured a homozygous NM_001297732.2:c.215T>G (p.Leu72Arg) (Fig. 1B). The variant is extremely rare (allele frequency of 0.00002, with no homozygotes in gnomAD.^45^ Family 3 carried compound heterozygous variants NM_001297732.2:c.328G>C (p.Ala110Pro) and c.893G>C (p.Arg297Pro), which were confirmed to be in trans (Fig. 1C). Both variants are exceedingly rare, with allele frequencies of 0.000001 and 0.00002, respectively, and had no homozygotes in gnomAD.^45^ All these missense variants co-segregated with the disease and were predicted to be deleterious by Polyphen-2 and CADD (CADD Phred > 20). The mtDNA analysis of the probands from Families 2 (2.II.1) and 3 (3.II.1) revealed no pathogenic mtDNA variants and showed that they carried the J2a2e and K1a haplogroups, respectively.

All the substitutions affected highly conserved amino acids across different vertebrate species (Fig. 1G). Given that no crystal structure is available for COX18, AlphaFold^49,50^ was used to determine the location and potential impact of the substituted residues. Leu72 lies in a short amphipathic helix (LH1) that faces the inner mitochondrial space, parallel to the inner mitochondrial membrane (Fig. 1E and F). The substitution by an arginine introduces a positive charge that might impact the hydrophobic interactions of that helix (Fig. 1F, *left*). Ala110 is at the matrix end of the first transmembrane helix (TM1) (Fig. 1E and F, middle), and the introduction of a buried proline in its place is predicted to be structurally damaging by Missense 3D-SB^51,52^ (Fig. 1F, bottom middle). The Arg297 residue is located at a loop immediately after the fifth transmembrane helix (TM5) (Fig. 1E) and shares hydrogen bonds with Ile241 in the third transmembrane domain (Fig. 1G, *top right panel*). The Arg297Pro substitution is predicted by Missense 3D-SB^51,52^ to damage the protein structure owing to disallowed conformations. Additionally, we hypothesize that the change might abolish the hydrogen bond shared with Ile241, a residue from the third transmembrane helix (TM3) (Fig. 1G, bottom right), which might be key for protein folding. This is supported by the FoldX^53^  *in silico* tool, which estimated that the Arg297Pro could result in a change of protein stability of 3.8 kcal/mol.

### *COX18* deficiency causes a predominant sensorimotor neuropathy phenotype

Detailed clinical features of all eight affected individuals are described in Table 1. The cardinal feature among all patients was progressive sensorimotor axonal polyneuropathy, with a variable onset ranging from 9 to 46 years of age. All patients presented initially with distal lower limb motor and sensory symptoms and, as the disease progressed, most of them slowly developed distal upper limb involvement. Most of the patients presented muscle weakness at disease onset, except for two siblings from Family 2, whose phenotypes are characterized predominantly by sensory loss in distal lower limbs. Muscle atrophy was prominent in the feet, in the tibialis anterior and the calf muscles. Two of the patients were dependent on mobility aids, such as a walking stick or wheelchair from middle age. All patients presented sensory loss, which followed a stocking and glove pattern. The sense of touch, proprioception and vibration were impaired, mostly in the lower limbs. One of the individuals (3.II.1) in addition developed, from her mid-thirties, pain in the feet and thighs accompanied by a band-like pressure sensation around the legs. Her affected sibling also complained about mild pain until the proximal segment of the calf. All patients suffered from foot deformities, usually in the form of pes cavus, but some of them also presented pes equinovarus and hammer toes. Most of the patients exhibited decreased or absent deep tendon reflexes in distal lower limbs, except for the patients from Family 3. More specifically, individual 3.II.1 had generalized brisk reflexes except for absent ankle reflexes, and as the disease progressed her gait became increasingly spastic and ataxic. Her sister, individual 3.II.2, had a similar presentation, with general hyperreflexia and absent ankle reflexes on examination.

**Table 1:** Clinical features of the index patients

General features	Family 1		Family 2				Family 3	
	II.1	II.2	II.1	II.2	II.3	II.4	II.1	II.2
Ethnicity/consanguinity	Serbian/yes		Egyptian/no				British/no	
Sex/AOO, years	F/∼10	M/∼10	M/40	M/46	M/28	M/30	F/9	F/14
Symptoms at onset	Weakness and wasting in DLL	Weakness and wasting in DLL	Numbness, weakness and wasting in DLL	Numbness, weakness and wasting in DLL	Intermittent numbness in DLL	DLL numbness, slipper slippage while walking	High feet arches, running difficulties	Ankle sprains and falls
Age at last examination, years	51	35	50	54	32	44	44	50
Main symptoms	Severe weakness in DLL and mild in DUL	Severe weakness in DLL and mild in DUL	Numbness, weakness in DLL and DUL	Weakness and numbness in DLL	Numbness, weakness and wasting in DLL	Weakness and wasting in DLL	Weakness in DLL and DUL, numbness in DLL	Weakness in DLL and DUL, numbness in DLL, ankle instability
Mobility	Wheelchair, walking stick from age 40 years	Independent	Independent	Independent	Independent	Independent	Spastic gait, independent	Walking stick from age 40 years
Clinical diagnosis	CMT2	CMT2	CMT2	CMT2	CMT2	CMT2	CMT2 with UMN signs	CMT2 with hyperreflexia
**Pyramidal and PNS**								
Strength DLL/DUL	0–1/3–4	0–1/5	0/4	0/4	2/5	1–2/5	1/4	0/3–4
Atrophy DLL/DUL^a^	++/+	++/+	++/+	++/+	++/+	++/+	++/+	++/−
Abnormal deep tendon reflexes	Achilles absent	Achilles absent	Achilles absent	Achilles absent	Achilles ↓	Achilles ↓	All ↑, Achilles ↓	All ↑, Achilles absent
Plantar responses^b^	Mute	Mute	Mute	Mute	Mute	Mute	Extensor	Mute
Foot deformities	Pes cavus equinovarus	Pes cavus	Pes cavus, hammer toes	Pes cavus, hammer toes	Pes cavus, hammer toes	Pes cavus, hammer toes	High arches	Bilateral foot triple arthrodesis in 20s
**Sensory**								
Touch deficit	+	+	+	+	+	+	NT	NT
Propioception/vibration deficit	+	+	+	+	+	+	+	+
Pain/temperature deficit	NT	NT	+	+	+	+	+	+
Neuropathic pain	−	−	−	−	−	−	Moderate	Mild
Brain MRI	Normal	−	−	−	−	−	Mild cerebellar atrophy	Normal
Other	Cervical dystonia; postural tremor of DUL; depression	Postural tremor of DUL	–	T2DM	–	–	Spasticity; sensory ataxia; spinal MRI: mild cervical spondylosis; mildly elevated serum CK; normal serum lactate	Spinal MRI: cervical spondylosis with C5–C6 exit foraminal stenosis; T2DM

AOO = age of onset; CK = creatine kinase; CMT2 = axonal Charcot–Marie-Tooth disease; DLL = distal lower limbs; DUL = distal upper limbs; F = female; M = male; NT = not tested; PNS = peripheral nervous system; T2DM = diabetes mellitus type 2; UMN = upper motor neuron; ↓ = reduced; ↑ = brisk.

^a^Muscle atrophy is reported as moderate (++), mild (+) or absent (−).

^b^Bilateral plantar responses are reported.

Nerve conduction studies (Table 2) revealed decreased compound muscle action potential (CMAP) in peroneal nerve recordings and, in some patients, also in the median nerve recordings. Sensory nerve action potentials (SNAPs) were undetectable in sural nerves in all patients, and ulnar SNAPs were unrecordable in five of eight of them. Motor nerve conduction velocity (MNCV) was within normal ranges in most patients. Some of the individuals exhibited slightly decreased MNCV which, in the context of low amplitudes, was attributed to axonal loss. In summary, electrophysiological studies indicated sensorimotor axonal neuropathy in all the patients.

**Table 2:** Electrophysiological studies of the index patients

Nerve	NCS	Family 1		Family 2				Family 3	
		II.1	II.2	II.1	II.2	II.3	II.4	II.1	II.2
Median	CMAP, mV	5.8	3.3	0.83	0.25	6.5	0.35	7.6	6.4
	MNCV, m/s	47.7	55.9	50	36	58.2	30.0	55	48
Peroneal	CMAP, mV	ND	ND	ND	0.45	4.53	ND	1.2	0.8
	MNCV, m/s				24.0	40.6		44	28
Ulnar	SNAP, µV	23.2	NP	20.8	ND	30.5	ND	ND	ND
	SNCV, m/s	40.0		28.6		26.8			
Sural	SNAP, µV	ND	ND	ND	ND	ND	ND	ND	ND
	SNCV, m/s								
Electrophysiological diagnosis		Sensorimotor axonal CMT	Sensorimotor axonal CMT	Sensorimotor axonal CMT	Sensorimotor axonal CMT	Sensorimotor axonal CMT	Sensorimotor axonal CMT	Sensorimotor axonal CMT	Sensorimotor axonal CMT

CMAP = compound muscle action potential; CMT = Charcot–Marie–Tooth disease; MNCV = motor nerve conduction velocity; NA = not applicable; NCS = nerve conduction studies; ND = not detected; NP = not performed; SNAP = sensory nerve action potential; SNCV = sensory nerve conduction velocity.

During the disease course, some patients developed additional symptoms beyond the peripheral nervous system. Affected individuals from Family 1 had upper limb tremor, and individual 1.II.1 developed cervical dystonia at 45 years of age (Supplementary Video 1), which was treated with botulinum toxin. Patients from Family 3 showed brisk deep tendon reflexes, and individual 3.II.1 had a particularly complex phenotype characterized by sensory ataxia, spastic gait and bilateral positive Babinski reflexes. Follow-up studies in this patient revealed mildly elevated serum creatine kinase 165 IU/l (normal range 26–140 IU/l), and brain and spinal MRI revealed mild cerebellar atrophy and cervical spondylosis without myelopathy. Serum lactate in individual 3.II.1 was normal. Her affected sibling, 3.II.2, showed a normal brain MRI, and spinal MRI showed cervical spondylosis with C5–C6 exit foraminal stenosis. Noteworthy, none of the patients presented other mitochondrially related conditions, such as cardiomyopathy, hepatopathy and tubulopathy.

### Splice variant c.435-6a>G leads to alternative splicing, resulting in the expression of an aberrant COX18 isoform

To evaluate the impact of the c.435-6A>G variant on splicing, cDNA T-LRS was carried out, where we opted to identify reliably potential splicing events triggered by the mutation and to quantify all *COX18* transcripts present (Fig. 2A and B). To do this, *COX18* cDNA was amplified from EBV-transformed lymphoblasts from the patients, their parents and unrelated controls. Primers were designed in exon 1 and 4 (Fig. 2A and Supplementary Table 1) to be able to distinguish all previously known and potentially novel *COX18* transcripts and their associated splicing events. Sequencing confirmed that the splice variant generated a new acceptor site in intron 2 that added five coding base pairs to exon 3 (Fig. 2A). The frameshift caused by this insertion generated a premature stop codon in the third exon of the canonical transcripts (Ensembl IDs ENST00000507544.3 and ENST00000295890.8). Accordingly, the mutant canonical transcripts represented a minor portion of the reads in patients and carriers, which suggests that they are most likely to be degraded by nonsense-mediated mRNA decay (NMD) (Fig. 2B). Interestingly, an alternative transcript skipping exon 2 represented as much as 66% of all the transcripts in the patients and 14%–27% in the heterozygotes (Fig. 2B). This transcript corresponds to an alternative transcript lacking exon 2 (ENST00000449739.6) that has a premature stop codon in exon 3 and is normally degraded by NMD. However, instead of being degraded, this transcript recovers its reading frame as a consequence of the splice variant. Canonical wild-type *COX18* transcripts (ENST00000507544.3 and ENST00000295890.8) totalled 86% of the transcripts in unrelated controls and 53%–61% in heterozygote carriers (Fig. 2B). In contrast, these canonically spliced transcripts constituted only 4%–8% in patients, suggesting that splicing leakage occurs at negligible levels. Another NMD transcript (ENST00000510031.1) with 4 bp added to exon 2 was observed in all samples in a minimal proportion, regardless of the genotype (data not shown).

The cDNA T-RLS findings were complemented by RT-qPCR experiments using primers located in the exon 5–6 junction and 3′ untranslated region. In this way, we quantified the total amount of the longest and predominant *COX18* transcripts in all individuals. As expected, the patients expressed lower levels of *COX18* in comparison to unrelated controls, and a similar trend was observed in comparison to the carriers, although it was not statistically significant (Supplementary Fig. 1).

### Mutant COX18 protein affects the assembly and stability of CIV subunits

COX18 is an assembly factor that translocates the C-terminus of MTCO2, a core subunit of CIV, across the inner mitochondrial membrane (IMM) into the inner mitochondrial space. Models in yeast and human cells have demonstrated that *COX18* knockout impairs MTCO2 insertion across the IMM and renders it unstable, ultimately leading to MTCO2 degradation.^54,55^ Mutant COX18 protein resulting from the transcript with frame recovery is predicted to be only 4 kDa smaller in molecular mass than the wild-type protein.

To evaluate the impact of the splice event on the quantity and function of COX18 and its partner MTCO2, we performed immunoblotting assays with patients’ lymphoblasts. Western blotting showed only one protein band corresponding to COX18 in the patients, suggesting that the mutant protein is stably expressed in the patients in comparable amounts to unrelated controls (Fig. 2C and Supplementary Fig. 2). However, MTCO2 levels were significantly decreased in patients compared with their heterozygous parents (Fig. 2C and D), suggesting that mutant COX18 might affect MTCO2 protein levels. In contrast, unrelated controls showed highly variable expression of MTCO2.

We also examined the ability of COX18 to translocate MTCO2 across the IMM using a proteinase K (PK) protection assay. The experiment showed that MTCO2 was PK sensitive and degraded in mitoplasts from the unrelated controls and the carriers, whereas in the mitoplasts of patients it was protected from degradation (Fig. 2E and F). These results point out that mutant COX18 has an impaired ability to insert MTCO2 C-terminus across the IMM, affecting its stability and expression level.

To assess the impact of mutant COX18 on the stability of CIV or other complexes, a representative subunit from each complex was immunoblotted (Fig. 2G). The experiment revealed decreased levels of CIV subunit MTCO1, whereas the subunits from other complexes did not show altered protein levels (Fig. 2H). This suggests that the variant might be associated with an isolated defect in CIV, but further follow-up studies are needed to confirm this.

### Splice variant c.435-6a>G impairs CIV activity and mitochondrial membrane potential

Considering that COX18 partial loss of function affects the stability of a core subunit of CIV, we evaluated whether this defect might impact its overall enzymatic activity. CIV is the final enzyme from the electron transport chain. It catalyses the oxidation of reduced cytochrome *c*, which generates water. This reaction is coupled with the transport of four protons across the IMM, contributing to the proton gradient that drives ATP synthesis.

To assess the impact of the variant on CIV activity, we performed an enzymatic assay that measures the rate of change in absorbance at 550 nm caused by the oxidation of cytochrome *c*. The assay revealed that patients had a lower CIV to CS enzymatic activity ratio in comparison to unrelated controls (Fig. 2I). In turn, decreased CIV activity could lead to reduced proton translocation through the IMM. Therefore, the mitochondrial membrane potential was measured using flow cytometry on patients’ lymphoblasts stained with TMRE, a dye that accumulates in cells with a hyperpolarized IMM. The TMRE signal was significantly lower in the cells from probands compared with carriers and unrelated controls, which indicates a decrease in mitochondrial membrane potential (Fig. 2J).

### *Drosophila melanogaster* COX18 knockdown model displays signs of neurodegeneration

**Figure 3:**
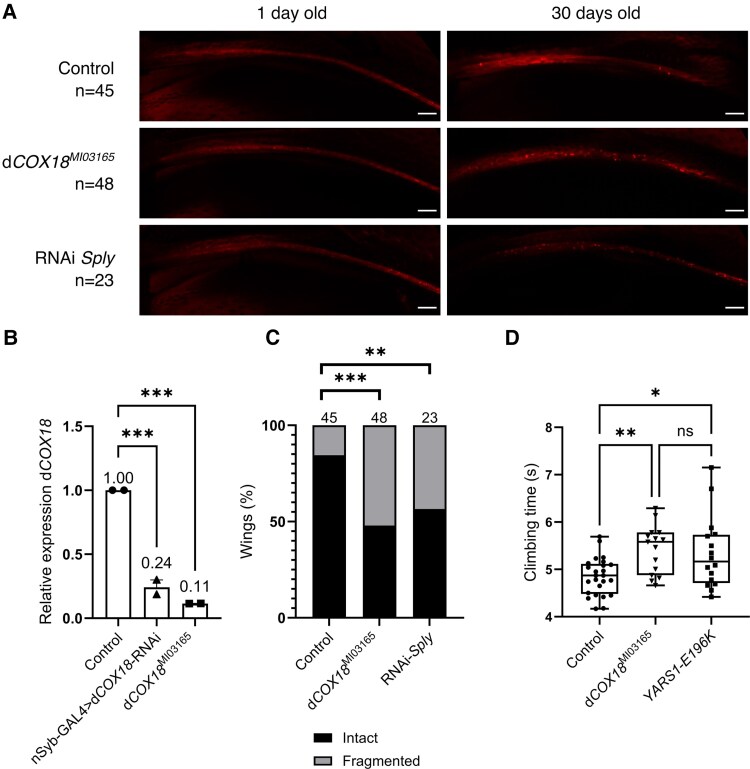
**Downregulation of *COX18*'s orthologue in *Drosophila*, d*COX18*, causes age-dependent axonal degeneration of sensory neurons and locomotor impairment.** (**A**) The nerve tract along the L1 wing vein was visualized by mCherry expression using dpr-GAL4 driver. Representative images are shown from 1- and 30-day-old flies from each genotype. RNAi Sply flies were used as a positive control. Flies expressing the driver alone were used as a negative control. (**B**) Quantification of d*COX18* RNA expression using RT-qPCR for control (*yw*), nSyb-driven RNAi d*COX18* pan-neuronal knockdown (nSyb-GAL4>RNAi-d*COX18*) and d*COX18^MI03165^* fly lines. Data are shown as the mean ± standard deviation (SD) (*n* = 2). (**C**) Quantification of the percentage of wings with axonal fragmentation at Day 30 (*n* = 23–48 per genotype). (**D**) Climbing performance of control (*yw*), d*COX18^MI03165^* and *YARS1-E196K* fly lines was assessed by measuring the climbing time of 10-day-old flies. Data are shown as the mean ± SD (*n* = 15–25, 10 groups of 10 flies tested for each phenotype). ***P < 0.001, **P < 0.01, *P < 0.05, ns = not significant.

COX18 is a key assembly factor of the mitochondria and, as such, has proved to be functionally conserved from fungi to mammals.^56,57^ The *COX18* orthologue in *D. melanogaster* (*CG4942*, d*COX18*) shares 61% similarity and 40% identity with the human protein (Supplementary Fig. 3).^58-60^ No fly phenotype has been reported to be associated with d*COX18* downregulation. To emulate the partial loss of function of *COX18* observed in the patients, we used a fly line with one copy of d*COX18* disrupted by the insertion of the transposon Minos-mediated integration cassette (MiMIC) within its coding sequence (d*COX18^MI03165^*). Notably, this leads to ∼90% reduction of d*COX18* mRNA levels in comparison to naïve flies (*yw*; Fig. 3B), whereas pan-neuronal downregulation of d*COX18* expression only led to ∼75% reduction (nSyb-GAL4>RNAi-d*COX18*; Fig. 3B). Furthermore, when crossing d*COX18^MI03165^* flies to obtain homozygous d*COX18*-deficient flies, we observed no offspring, suggesting that the complete loss of d*COX18* in the homozygous null flies is not viable. Therefore, we chose the d*COX18^MI03165^* fly line with severely reduced d*COX18* expression as a biologically relevant model for studying COX18 partial deficiency.

We then assessed whether the d*COX18^MI03165^* flies displayed behavioural and histopathological signs of neurodegeneration. Initially, we performed a wing degeneration assay to assess whether d*COX18* partial loss is associated with axonal degeneration. We evaluated the integrity of the long axons of the chemosensory neurons innervating the wing margin bristles by expressing a fluorescent red mCherry protein (UAS-mCD8::chRFP) in the neuronal membrane (dpr-GAL4 driver) (Fig. 3A). No difference was observed between the fly lines carrying different genotypes on the first day after eclosion. Nonetheless, 30-day-old d*COX18^MI03165^* flies exhibited prominent axonal fragmentation compared with the control fly line (Fig. 3A and C). This degenerative phenotype was comparable to the effect caused by downregulation of *Sply*, the *Drosophila* orthologue of *SGPL1* that causes axonal CMT in humans.^43^ In addition, a negative geotaxis climbing assay was conducted to evaluate the locomotor performance of the d*COX18^MI03165^* flies (Fig. 3D). In this test, the d*COX18^MI03165^* flies presented a slower climbing speed in comparison to the controls (Fig. 3D). The climbing performance of d*COX18^MI03165^* flies was similar to the locomotion deficit presented by another well-established *Drosophila* model expressing a CMT-causing variant in the *YARS1* gene.^61^

## Discussion

This study provides genetic and functional evidence to support *COX18*, a nuclear-encoded mitochondrial assembly factor, as a novel CMT gene candidate. Biallelic missense and splice variants in *COX18* were identified in three families with autosomal recessive axonal CMT. *In silico* predictions and *in vitro* and *in vivo* studies suggest that *COX18* partial loss of function is the underlying disease mechanism. Congruently, downregulation of the *COX18* homologue in *Drosophila* replicates key features of neurodegeneration, such as locomotor impairment and axonal degeneration of sensory neurons.

After screening in-house and external CMT cohorts, we have found in total eight patients from three families with biallelic variants in *COX18*. All patients showed sensorimotor axonal polyneuropathy that primarily affects the distal lower limbs. They presented with muscle weakness and atrophy accompanied by foot deformities. The motor impairment significantly affected the ambulation of the patients from Families 1 and 2, who were dependent on mobility aids. All individuals experienced sensory loss, which was more predominant amongst the affected members from Family 3. Electrophysiological studies revealed axonal degeneration of motor and sensory nerves from upper and lower limbs. This is compatible with the literature, because the most common type of neuropathy observed in mitochondrial disorders is axonal.^62-64^

Peripheral neuropathy occurs in approximately one-third of the patients with mitochondrial diseases.^65-69^ Given that mitochondrial function is essential for many tissues and systems, mitochondrial peripheral neuropathy usually occurs together with other neurological and extra-neurological manifestations, such as encephalopathy, myopathy, cardiac disease and renal dysfunction.^64,65,67^ In some cases, neuropathy can be the only manifestation at onset, but subsequently, other tissues might become affected as the disease progresses.^14,65,70^ Likewise, some of the patients reported here developed CNS symptoms during the disease. For instance, Patient 1.II.1 developed cervical dystonia in her 40s, three decades after the disease onset. Family 2 seems to be the exception, because all four affected siblings do not show any CNS manifestation. Nevertheless, their peripheral neuropathy has a late onset (∼40s), and it is still plausible that additional symptoms might develop at later stages of the disease.

COX18-related neuropathy showed notable inter- and intrafamilial phenotypic variability. Disease onset varied considerably between families, from late childhood to middle adulthood. This clinical heterogeneity might be attributable to the distinct impact of each variant on COX18 function or the differences in genetic background and epigenetics between the patients. Mitochondria are highly dynamic organelles that can alter their mass, shape and number to compensate metabolic insults.^71^ Moreover, owing to the dual genetic origin of mitochondrial proteins, the interaction between nuclear DNA and mtDNA might modulate the penetrance and severity of mitochondrial disease.^72-74^ For example, mtDNA variants have been shown to significantly influence the cardiomyopathy phenotype of mice caused by a mutation in a mitochondrial nuclear DNA gene.^75^ In this study, we did not identify deleterious mtDNA variants that could explain the clinical differences observed, but the probands from each family carried a different mitochondrial haplogroup, which might modify the disease expresion. Finally, environmental modifiers might also play a role in determining the severity and progression of mitochondrial disorders, as observed in mitochondrial optic neuropathies.^76,77^

In this study, we have identified four different *COX18* variants: three missense and one splice variant. All were segregated with the disease in each family and were either novel or extremely rare in public databases. All three missense variants perturbed conserved residues in different domains of the protein. *In silico* predictions suggest that they are likely to be deleterious. The p.Ala110Pro and p.Arg297Pro variants are predicted to disrupt the protein structure. Although p.Leu72Arg might not affect COX18 structure, it introduces a positive charge in the LH1 amphipathic helix that might affect its function. This domain has been proved to be essential for the insertase function of proteins from the same family (Oxa1/YidC/Alb3) and is thought to destabilize the lipid bilayer and facilitate the release of the inserted protein into the membrane.^78,79^ Based on this evidence, these missense variants meet PM2, PP3 and PP1 criteria (variants of unknown significance) from the American College of Medical Genetics and Genomics (ACMG) guidelines.^80^ Functional validation of the missense variants is necessary to understand the precise effect of the predicted structural perturbations on COX18 activity.

We studied in detail the functional effect of the splice variant c.435-6A>G. The variant reduces the expression of the canonical transcript to negligible levels and, at the same time, generates an alternatively spliced product missing exon 2. This transcript is the predominant isoform in the patients, whereas the canonical transcript is marginally expressed. Thus, we attribute the COX18 protein observed in the patients to the expression of a stable but partly functional mutant protein. According to COX18 *in silico* structural models,^49,50^ the loss of exon 2 would disrupt a helical hairpin (M1) (Fig. 1E) that is well conserved in all translocases from the Oxa1/YidC/Alb3 family.^81^ Consistent with our findings, deletions in the same region in the *COX18* orthologue, *yidC*, in *Bacillus subtilis* and *Escherichia coli* do not affect the protein stability but significantly impair its translocase function.^79,82^ It is hypothesized that this dynamic and flexible hairpin is in charge of substrate recruitment.^81,83^ Likewise, our results highlight the importance of this domain, because the aberrant COX18 isoform identified shows a reduced ability to translocate MTCO2 C-terminus, probably owing to difficulties in its recruitment. Thus, based on the evidence provided in this study, the c.435-6A>G variant can be classified as pathogenic according to the ACMG criteria (PS3, PM2, PM4, PP3 and PP1).^80^

MTCO2 is a core subunit of CIV, which accepts the electrons from cytochrome *c* through the copper centre in its C-terminal and transfers them across the complex to produce water. Patients with the homozygous splice variant showed decreased levels of the substrate of COX18, MTCO2, in comparison to their heterozygous parents, who share a similar genetic background. Yet, this difference was not observed when compared with five unrelated controls, who showed high interindividual variability in MTCO2 expression, in accordance with previous reports.^84,85^ Crucially, despite comparable MTCO2 levels to controls, the PK protection assay demonstrated that MTCO2 in patient mitochondria is protected from protease degradation. This indicates that the protein is not properly inserted into the IMM, preventing the C-terminal domain from performing its essential role in electron transfer, and therefore, compromising the function of CIV as a whole. Furthermore, previous studies in *COX18* knockout HEK293T cells demonstrated that COX18 translocase activity is necessary for the post-translational stability of MTCO2.^55^ The decreased MTCO2 protein levels seen in the patients relative to the parents can therefore be attributable to this defect in the insertion and folding of MTCO2, which renders it unstable and prone to degradation. The COX18 deficiency observed in the patients was also associated with decreased levels of MTCO1, suggesting a deleterious effect on the stability of another CIV subunit. In turn, these defects were correlated with reduced CIV enzymatic activity and impaired mitochondrial membrane potential. Taken together, we demonstrate that the splice variant affects the chaperone and translocation function of COX18, which ultimately compromises the role of CIV as part of the mitochondrial electron transport chain.

*COX18* is expressed in multiple tissues, with the highest expression in EBV-transformed lymphoblasts, adrenal glands and peripheral nerves.^86^ To assess the susceptibility of neurons to *COX18* downregulation in a whole organism, we studied a d*COX18* deficient fly model. The d*COX18^MI03165^* fly displayed signs of neurodegeneration, including age-dependent axonal degeneration of sensory neurons in the wing and locomotor impairment. These results were comparable to the phenotypes observed in previously published CMT fly models, studying mitochondrial or non-mitochondrial CMT-associated genes, which exhibit axonal degeneration and climbing defects as the d*COX18^MI03165^* fly.^16,43,44,87^ A similar locomotor impairment has been described in a fly knockdown model of *COA7*, another assembly factor of CIV that has been reported to cause CMT.^16^ Additional functional studies on *in vivo* and *in vitro* neuronal models are required to understand the susceptibility of the motor and sensory neurons to the loss of COX18 and to CIV deficiency in general.

It was not possible to obtain a homozygous null d*COX18* fly, which might suggest that complete knockout of d*COX18* is not viable. Although full knockout of this gene might be lethal, flies expressing ∼10% of *COX18* orthologue were viable and fertile and demonstrated neurodegenerative phenotypes. Likewise, *COX18*^−/−^ mice exhibit embryonic growth retardation, eventually leading to prenatal or preweaning lethality.^88^ It is worth noting that some of these mice show abnormalities in neural tube closure. Altogether, a complete COX18 loss of function seems to be incompatible with life in different species. Therefore, we hypothesize that the variants reported in this study are probably hypomorphic and only partly reduce COX18 function or protein levels.

Despite an earlier study that screened a cohort of patients with CIV deficiency for *COX18* pathogenic variants,^89^ the gene had not been linked to any human disease until recently.^24,25^ The first report of COX18-related pathology described a patient with neonatal encephalo-cardiomyopathy and CIV deficiency who carried a homozygous NM_001297732.2:c.667G>C p.(Asp223His) variant.^24^ Likewise, a recent study reported a *COX18* NM_173827.4:c.598G>A(p.Gly200Ser) variant in a patient who developed, from 7 months of age, severe and rapidly progressive motor impairment, resembling spinal muscular atrophy, with dysarthria and oculofacial apraxia.^25^ Remarkably, both patients also presented axonal peripheral neuropathy. Additionally, an ES study identified, through homozygosity mapping, *COX18* biallelic variants as a candidate cause for non-syndromic hearing loss, although functional evaluation was not conducted.^90^ Thus, exhaustive phenotyping of additional patients is needed to ascertain the clinical spectrum of COX18-related conditions and further support its role in CMT pathogenesis.

Our findings on COX18-related CMT neuropathy illustrate that in the rare cases where peripheral neuropathy is the main or only clinical feature of an underlying mitochondrial disorder, it is likely to overlook the mitochondrial origin.^65,70^ The underlying mitochondrial aetiology can be suspected through histochemical, biochemical and neuroimaging studies. However, no single biomarker is sensitive enough to confirm the diagnosis completely, and the lack of abnormal biomarkers does not exclude a mitochondrial dysfunction.^76,91^ Likewise, mitochondrial biomarkers, including serum lactate and MRI, did not show consistent findings suggestive of a mitochondrial pathology in our patients. The application of unbiased genetic testing was crucial for establishing the correct aetiology. Likewise, using genetic approaches, several studies in the past decade have found different proteins of the mitochondrial respiratory chain to be implicated in the pathogenesis of CMT.^16-22^ Thus, our findings, together with these reports, stress the importance of screening mitochondrial genes as part of the diagnostic work-up of patients with CMT, with or without a multisystemic clinical presentation.

Among them, biallelic variants in genes encoding subunits or assembly factors of CIV (particularly *SURF1*, *COA7*, *COX6A1* and *COX20*) have been found to cause CMT.^16-22^ Strikingly, some of these genes encode proteins that are also involved in the translocation and maturation of MTCO2. For example, COX20 plays a role as the counterpart of COX18 by translocating the N-terminus of MTCO2.^20,92,93^ SCO2 is a metallochaperone that adds copper to the C-terminus of MTCO2 once COX18 translocates it into the inner mitochondrial space.^17^ What is more, a missense deleterious variant in the mitochondrial gene that encodes MTCO2, the substrate for COX18, has been reported to cause late-onset cerebellar ataxia, axonal peripheral neuropathy and tremor.^94^ Interestingly, some of these genes have also been reported to cause severe multisystemic diseases, such as Leigh syndrome (*SURF1*) and cardioencephalomyopathy (*SCO2*).^95,96^ These reports, together with the findings of the present study, underscore the role of CIV dysfunction in the pathogenesis of CMT. Further research is needed to understand what makes peripheral neurons particularly susceptible to CIV deficiency and to explain its broad clinical spectrum.

## Conclusion

In conclusion, we have provided genetic and functional evidence to support *COX18* as a new candidate gene for autosomal recessive axonal CMT. These findings underscore the importance of peripheral neuropathy in the spectrum of mitochondrial disorders, warranting the screening of mitochondrial genes in the diagnostic follow-up of CMT patients with or without CNS features. Our results also recommend the application of next-generation sequencing techniques in the diagnosis of non-syndromic neuropathies of mitochondrial aetiology. Moreover, our study provides further evidence to support the critical role of the hairpin domain of COX18 for its translocase activity. Finally, we draw special attention to the impact of mitochondrial CIV deficiency in the pathogenesis of CMT.

## Supplementary Material

awaf300_Supplementary_Data

## Data Availability

Experimental data generated during this study can be shared by the corresponding author on reasonable request from any qualified investigator.
